# Polycarbonate/Titania Hybrid Films with Localized Photo-Induced Magnetic-Phase Transition

**DOI:** 10.3390/nano11010005

**Published:** 2020-12-22

**Authors:** Shuta Hara, Sei Kurebayashi, Genza Sanae, Shota Watanabe, Takehiro Kaneko, Takeshi Toyama, Shigeru Shimizu, Hiroki Ikake

**Affiliations:** Department of Materials and Applied Chemistry, College of Science and Technology, Nihon University, 1-8-14 Kandasurugadai, Chiyoda-ku, Tokyo 101-8308, Japan; hara.shuta@nihon-u.ac.jp (S.H.); sei.kurebayashi@polymer.chem.cst.nihon-u.ac.jp (S.K.); genza.sanae@polymer.chem.cst.nihon-u.ac.jp (G.S.); syouta.watanabe@polymer.chem.cst.nihon-u.ac.jp (S.W.); kaneko.takehiro@nihon-u.ac.jp (T.K.); touyama.takeshi@nihon-u.ac.jp (T.T.); shimizu.shigeru@nihon-u.ac.jp (S.S.)

**Keywords:** networks, nanocomposites, polycarbonates, magnetic polymers, oligomers, Small Angle X-ray Scattering (SAXS), synthesis

## Abstract

Materials that exhibit the photo-induced magnetic-phase transition of titania are receiving significant attention because they can be easily switched between diamagnetism and paramagnetism by UV irradiation. However, it is difficult to store photo-induced titanium (Ti^3+^) in air because of its easy oxidation upon oxygen exposure. In this study, titania/polycarbonate hybrid films were prepared using linear 1,6-hexanediol (PHMCD), cyclic 1,4-cyclohexanedimethanol (PCHCD), or their copolymerized carbonate oligomers using the sol–gel method. The oxygen permeability of the hybrid film decreased as the ratio of the ring structure increased by a factor of approximately 32 from PHMCD with only the chain structure to PCHCD with only the ring structure. These hybrid films can generate Ti^3+^ under a UV irradiation of 250 W for 2 h, and the difference in oxygen permeability significantly affected the lifetime of the Ti^3+^ by a factor of up to 120. In addition, the tensile tests and IR measurements demonstrated that UV irradiation had little effect on the mechanical intensity and matrix chemical structure. Moreover, the magnetic susceptibility of Ti^3+^ present in PCHCD was confirmed to be 6.2 (10^−3^ emu/g(titania)) under an external magnetic field of 5 T induced using a superconducting quantum interference device.

## 1. Introduction

The method of adding inorganic components to a polymer matrix is one of the effective approaches for the multifunctionalization of polymer materials [1,2,3,4,5,6]. In particular, inorganic components with photochromic properties are essential for the development of polymer materials with photosensitivity. Various inorganic materials, such as titania and tungsten, have been reported as inorganic components possessing photochromic properties. Particularly, in recent years, Ti^3+^ that is generated by irradiating Ti^4+^ with UV has received significant interest because it behaves as a paramagnetic material with unpaired electrons [7,8,9,10,11,12,13,14]. This mechanism can be potentially used to develop magnetic materials that respond to stimuli or facilitate the free patterning of magnetization. However, the lifetime of photo-induced Ti^3+^ in the atmosphere is difficult to control because it is easily oxidized by oxygen [15,16,17,18,19]. Therefore, to reproduce the magnetic transition of Ti^3+^ under atmospheric conditions, it is necessary to control oxygen permeability of the polymer matrix.

To develop a hybrid material that can undergo photochromic reaction, it is important that the polymer matrix exhibits not only controllable oxygen permeability but also mechanical strength and photochemical stability, all of which makes the material applicable for practical use. In the case of hybrid materials, it is important that their inorganic domains are uniformly dispersed on a nanoscale in order to obtain mechanical strength [20,21,22,23,24,25]. Such nanoscale dispersions can be achieved using the sol–gel method. Generally, this method is carried out using a polymer modified using silica alkoxide in the main chain or at the terminal and metal alkoxide [26,27,28,29,30,31,32,33,34]. Previously, we have investigated a polycarbonate/titania hybrid system composed of linear 1,6-hexanediol (PHMCD) and cyclic 1,4-cyclohexanedimethanol (PCHCD) as non-toxic alternatives to bisphenol A [35]. Using the sol–gel method, titania hybrid films were prepared with silane group-functionalized oligo-polymers and tetraisopropyl orthotitanate. These materials exhibited high transparency with 40 wt% titania dispersed on the nanoscale in the polymer matrix. Moreover, the glass transition temperature (*T*_g_) and refractive index were observed to depend not only on the molecular weight of the oligo-polymer but also on the ring structure in the main chain. In particular, the difference in *T*_g_ due to the packing effect of the ring structure is closely related to the airtight characteristic of the hybrid polymer. Therefore, it is reasonable to consider that these polycarbonates provide the abovementioned conditions, including the regulation of oxygen permeability.

**Figure 1:**
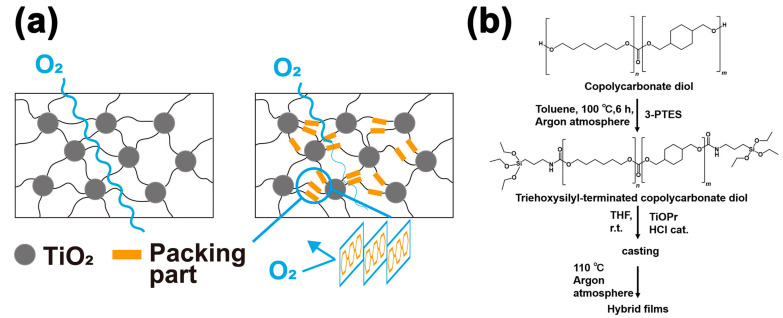
(**a**) Schematic diagram of the effect of the packing part constructed by the ring structure on oxygen permeability. (**b**) A modification reaction of isocyanatopropyltriethoxysilane (3-PTES)) of the polycarbonate diol and sol-gel reaction for preparing to titania contained polycarbonate films.

In this study, we report that the ring structure of polycarbonate can regulate the oxygen permeability, simultaneously, not only maintain mechanical strength and transparency but also be controlled the magneto-optical switching function of titania. This study demonstrates that the ring structure of polycarbonate generates a packing part and prevents oxygen from entering the 3D network (Figure 1a). To our knowledge, no study has focused on the relationship between the main chain structure and the oxygen permeability of hybrid materials. Titania polycarbonate hybrid materials were prepared by the sol-gel reaction with 40 wt% titanium isopropoxide (TiOPr) loading and terminal triethoxysilane modification with PHMCD, PCHCD, and oligomers with different copolymerization ratios (Figure 1b) [35]. The dispersibility of titania in these materials was evaluated using ultraviolet–visible (UV–Vis) measurements and small angle X-ray scattering (SAXS), and it was shown that titania was uniformly dispersed at the nanoscale. In addition, the oxygen permeability test showed that the oxygen permeability decreased as the ring structure increased, and the difference was approximately 32 times. Ti^3+^ can be produced using these hybrid materials through UV irradiation at 250 W for 2 h. Despite the intense UV irradiation, the decrease in transparency and mechanical strength was minimal after Ti^3+^ disappeared. The lifetime of Ti^3+^ was examined in detail using electron spin resonance (ESR) measurements, and the lifetime of Ti^3+^ increased with decrease in the oxygen permeability, and the difference reached approximately 120 times. The magnetic susceptibility of Ti^3+^ present in PCHCD was confirmed to be 6.2 (10^−3^ emu/g(titania)) under an external magnetic field of 5 T induced using a superconducting quantum interference device (SQUID). Moreover, PCHCD was demonstrated to partially produce Ti^3+^ ions through the combination of photomask and UV irradiation.

## 2. Experimental

### 2.1. Materials

The oligo polyhexamethylene carbonate diols (PHMCD (*n*:*m* = 1:0) and PCHCD (*n*:*m* = 0:1)) and the oligo copolymerized (n = 1,6-hexanediol and m = 1,4-cyclohexanedimethanol) carbonate diols (coPCD 13 (*m*:*n* = 1:3), coPCD 11 (*m*:*n* = 1:1), coPCD 31 (*m*:*n* = 3:1) ), whose structures are described in Figure 1b, were obtained from Ube Industries, Ltd., Minato-ku, Toyko, Japan. Their respective number-average molecular weight (Mn) and the corresponding OH number were as follows: PHMCD: Mn = 1009, 110 mg KOH/g; coPCD 13: Mn = 916, 125 mg KOH/g; coPCD 11: Mn = 899, 125 mg KOH/g; coPCD 31: Mn = 934, 125 mg KOH/g; and (PCHCD): Mn = 1025; 110 mg KOH/g. Toluene, tetrahydrofuran (THF), and n-hexane were purchased from Kanto Chemical Co., Inc.,Chuo-ku, Tokyo, Japan titanium isopropoxide (TiOPr) was purchased from Nippon Soda Co., Ltd., Chiyoda-ku, Tokyo, Japan. and isocyanatopropyltriethoxysilane (3-PTES) was purchased from Tokyo Chemical Industry Co., Ltd., Chuo-ku, Tokyo, Japan.

### 2.2. Synthesis of TEOS-Modified Oligo Polyhexamethylene Carbonate Diols

The synthesis of TEOS-modified oligo polyhexamethylene carbonate diols was performed as follows (Figure 1b) [35]. Twenty-four milli mol of dried each PCDs (PHMCD, coPCD 13, coPCD 11, coPCD 31, PCHCD ) 2.5 times mol of 3-PTES relative to PCDs, and 100 mL of toluene were mixed in a 500 mL three-necked flask. The mixture was reacted at 100 °C for 6 h under an argon atmosphere. After the completion of the reaction, the mixture was precipitated with n-hexane to remove unreacted substances and vacuumed for 24 h to obtain the dried oligo-polymer samples. These samples were denoted as ET-PHMCD, ET-coPCD 13, ET-coPCD 11, ET-coPCD 31, and ET-PCHCD. The ^1^H-NMR spectrum of each oligo-polymer in deuterated chloroform is shown in Figures S1–S5.

### 2.3. Preparation of Polycarbonate/Titania Hybrid Films

Two point five gram of the each ET-PCDs (ET-PHMCD, ET-coPCD 13, ET-coPCD 11, ET-coPCD 31, and ET-PCHCD) were dissolved in 80 mL of dehydrated THF. TiOPr (40 wt% based on each ET-PCDs) was dropwise added into this solution. Subsequently, 25 μL of 1.0 M HCl was slowly dropwise added into the mixture and stirred at 20 °C for 15 min. The mixture solution was cast in a Glass petri dish. The mixture dried up for 3 days at 25 °C. Heat treatment of casted films were carried out at 80 °C for 24 min in an argon atmosphere to obtain a hybrid film. According to the ET-PCD used, the hybrid films were denoted as PC0 (ET-PHMCD), PC25 (ET-coPCD 13), PC50 (ET-coPCD 11), PC75 (ET-coPCD 31), and PC100 (ET-PCHCD) (Table 1).

### 2.4. Characterization

Tensile tests of the polycarbonate/titania hybrid films were performed at a tensile speed of 30 mm/min using IM-20 (INTESCO, Inc., Matsudo-City, Chiba, Japan). Measurement of UV–Vis near-infrared spectrophotometer was carried out at 25 °C using V-670 (JASCO Corporation, Hachioji-City, Tokyo, Japan). Thickness correction for UV–Vis near-infrared measurement was carried out using the following formula based on the Beer–Lambert law:

$$\begin{array}{rr} T_{1} & =\exp\left(-\mu t_{1}\right)\\ T_{2} & =\exp\left(-\mu t_{2}\right)\\ T_{1} & =\exp\left(\ln\frac{T_{2}}{100}\times \frac{t_{1}}{t_{2}}\right)\times 100 \end{array}$$

where $T_{1}$ is the transmittance after thickness correction (%), $T_{2}$ is the measured transmittance (%), $t_{1}$ is the thickness correction value (cm, reference thickness: 0.01 cm), $t_{2}$ is the measured sample thickness (cm), and μ is the linear absorption coefficient. SAXS measurements were performed using SAXSess (Anton Paar). The applied voltage and current of the X-ray source were 50 kV and 40 mA, respectively, and the CuKα line (*λ* = 0.154 nm) was selected. The camera length was 265 mm, and the measurement temperature was 30 °C. The scattering intensity was corrected by slitting using the Glatter method after correction, such as normal transmittance, and converted into the scattering intensity *I*(*q*) using the moving slit method.

*q* = (4π/λ)sin(*θ*/2).

Here, *q* is the scattering vector, and *θ* is the scattering angle. UV irradiation experiment of the polycarbonate titania hybrid films was performed at 250 W using a DGM2501A-01 (Sun Energy Corporation, Minoh-City, Osaka, Japan). Dynamic thermomechanical analysis (DMA) of the polycarbonate/titania hybrid films was carried out in the tension mode at a frequency of 1 Hz, a temperature range of −100–200 °C, with a heating rate of 5 °C/min, under a nitrogen atmosphere using DMS 6100 (Hitachi High Technologies, Minato-ku, Tokyo, Japan). Differential scanning calorimetry (DSC) measurements were performed with a temperature range of −80 to 200 °C, a heating rate of 5 °C/min, under a nitrogen atmosphere using DSC 6100 (Seiko Instruments Inc. , Chiba-City, Chiba, Tokyo). Measurement of infrared (IR) spectra of the polycarbonate/titania hybrid films was carried out using a Fourier-transform infrared spectrometer, FT/IR-6600 (JASCO.Co. Hachioji-City, Tokyo, Japan). Measurement of electron spin resonance (ESR) spectra of titania were carried out at 20 °C and an X-band frequency of 9.23 GHz. using JES-RE2X (JEOL, Akishima-City, Tokyo, Japan). The g value, which represents the electron orbital state peculiar to a substance, is calculated by the following formula:

*g* = *hν*/*βH*_0_,

where *h* is Planck’s constant, *ν* is microwave frequency, *β* is Bohr magneton, and *H*_0_ is the magnetic field. Oxygen permeability tests were conducted at 23 °C according to JIS K7126-1:2006 (for gas permeability) using a pressure sensation method. Magnetization measurements were performed on a MPMS-5S SQUID (Quantum Design, Toshima-ku, Tokyo, Japan).

## 3. Results and Discussion

### 3.1. Characterization of Titania Hybrid Films

**Figure 2:**
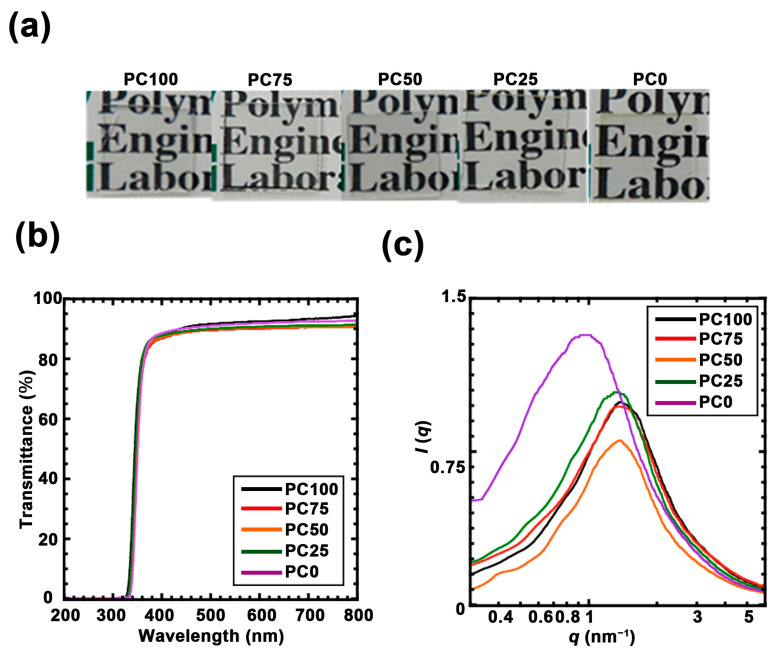
(**a**) Photographs of each polycarbonate/titania hybrid film. (**b**) UV-vis spectra of each polycarbonate/titania hybrid film: PC100 (black line), PC75 (red line), PC50 (orange line), PC25 (green line) and PC0 (purple line). (**c**) Small angle X-ray scattering (SAXS) profile of polycarbonate/titania hybrid film: PC100 (black line), PC75 (red line), PC50 (orange line), PC25 (green line), and PC0 (purple line).

Polycarbonate/titania hybrid films containing 40 wt% titania with different oligomers were successfully synthesized using the sol–gel method (Figure 2a). Although the magnetic susceptibility of the hybrid polymer can be increased according to the titania content, the mass ratio of polycarbonate/titania was maintained at 40 wt% because the cast samples containing more titania exhibited excessive shrinkage. Figure 2b shows the UV–Vis spectra for the polycarbonate/titania hybrid films after correction to 100 μm thickness. All the hybrid films exhibited high visible-light transparency (>90%) and blocked light at wavelengths below 350 nm due to absorption by titania. To evaluate the dispersibilities of the titania domains in each hybrid film, SAXS measurements were performed (Figure 2c). The intensity *I*(*q*) was plotted against the scattering wave vector *q* [=(4π/*λ*)sin (*θ*/2)], where *λ* is the X-ray wavelength (α; *λ* = 0.154 nm), and *θ* is the scattering angle. A peak derived from the titania domain was observed in all the hybrid films. PC0 has a peak at a lower q value than other samples due to the effects of extremely small amounts of crystal scattering. The distance between the titania domains (*R*_d_) was approximated using the Bragg’s Equation (1) (Table 2).

*R*_d_ = 2π/*q*(max) (1)

**Figure 3:**
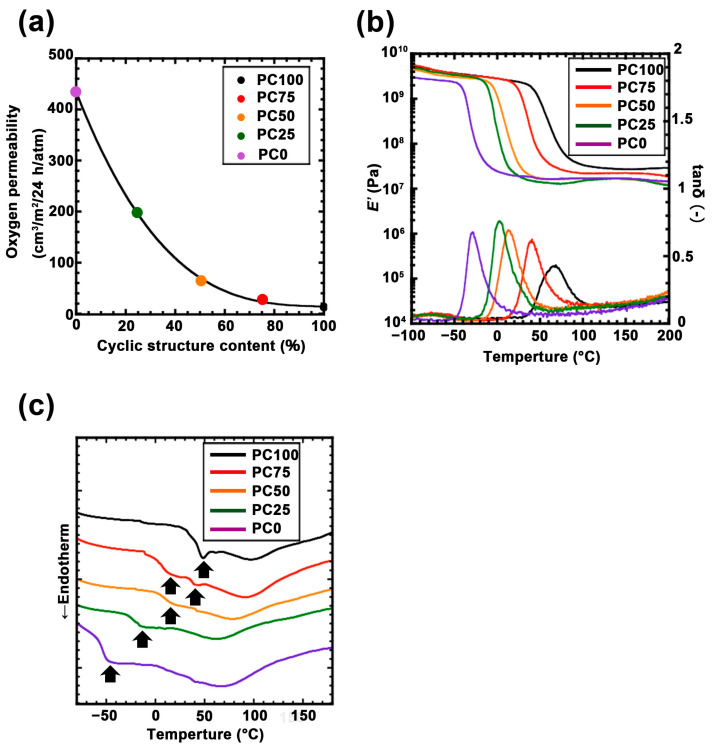
(**a**) Oxygen permeability test of each polycarbonate/titania hybrid film: PC100 (black dot), PC75 (red dot), PC50 (orange dot), PC25 (green dot) and PC0 (purple dot). (**b**) Dynamic thermomechanical analysis (DMA) curve for the polycarbonate/titania hybrid film: PC100 (black line), PC75 (red line), PC50 (orange line), PC25 (green line), and PC0 (purple line). (**c**) Differential scanning calorimetry (DSC) curve for each polycarbonate/titania hybrid film: PC100 (black line), PC75 (red line), PC50 (orange line), PC25 (green line), and PC0 (purple line).

The results confirmed that titania was finely dispersed at approximately 5 nm intervals in all the polycarbonate matrices. Moreover, *R*_d_ shrank as the main chain of the ring structure increased. Previous studies have demonstrated that these polycarbonates can be assumed to be Gaussian chains, and *R*_d_ reflects the size of the inorganic domain [33,35]. This SAXS profile suggested that the titania domain size is not affected by the cyclic structure of polycarbonate. The relationship between oxygen permeability and the ratio of the ring structure is shown in Figure 3a. Oxygen permeability increases logarithmically as the ring structure decreases. The ratio between the highest oxygen permeability (PC0, 433.0 cm^3^/m^2^/24 h/atm) and the lowest oxygen permeability (PC100, 13.3 cm^3^/m^2^/24 h/atm) was approximately 32. This trend has an inflection point at 50% of the ring structure. This result suggests that the ring structure is required over 50% to impart the packing part which is effective for oxygen permeability to the hybrid film. The DMA and DSC measurements were performed to examine the thermodynamic effects of these packing structures. From the DMA results (Figure 3b), it can be observed that a rubbery plateau was observed in all the samples, indicating that these samples developed a 3D network structure with titania. The *E*’ value in the plateau region increased as the ratio of ring structures increased. The *T*_g_ of each hybrid film (derived from the topmost peak region of tan δ) also shifted higher as the ratio of the ring structure increased (Table S1). Assuming that the cross-link density of the hybrid film is constant, these results indicate that the ring structure enhanced the intermolecular force between the polymer main chains. In addition, the results of the DSC analysis of the hybrid films are shown in Figure 3c. In PC100, a sharp peak derived from the glass transition temperature was observed at approximately 50 °C. Further, the peak derived from the glass transition temperature of PC0 was high. These temperature regions were correlated with the temperature derived from the topmost peak region of tan δ in DMA. This broad peak decreased as the linear structure decreased, and two peaks were observed at PC75. The separation of these glass transition temperatures indicated the phase separation of the linear structure and the cyclic structure. Moreover, it supported the hypothesis that the difference in oxygen permeabilities is due to the packing effect of the ring structure because the intermolecular force increased with the increasing contact area between the molecules.

### 3.2. Generation of Ti^3+^ by UV Irradiation 

**Figure 4:**
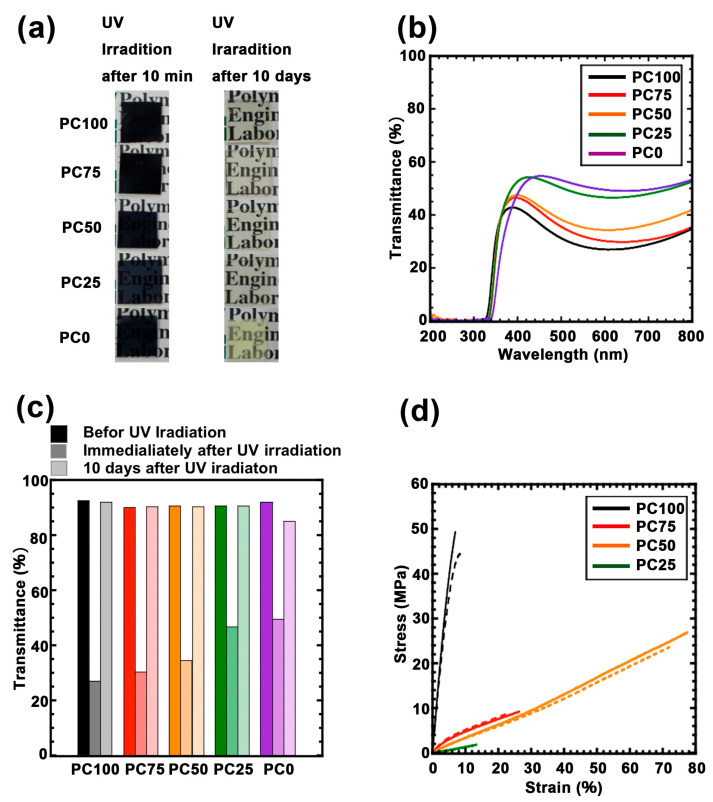
(**a**) Photograph of each polycarbonate/titania hybrid films immediately after UV irradiation and 10 days after UV irradiation. (**b**) UV-vis spectra of each polycarbonate/titania hybrid films immediately after UV irradiation PC100 (black line), PC75 (red line), PC50 (orange line), PC25 (green line), and PC0 (purple line). (**c**) Transmittance profile of each polycarbonate/titania hybrid film at 600 nm after UV irradiation: a lowest brightness is the films before UV irradiation, a medium brightness is UV irradiated films after 2 h, a highest brightness is UV irradiated films after 10 days, PC100 (black bars), PC75 (red bars), PC50 (orange bars), PC25 (green bars), and PC0 (purple bars). (**d**) Stress-strain curve for each hybrid film: the lines are before UV irradiated films, the dotted line is UV irradiated films after 10 days, PC100 (black bars), PC75 (red bars), PC50 (orange bars), PC25 (green bars), and PC0 (purple bars).

Figure 4a shows the images of the films after UV irradiation for 2 h. The polycarbonate titania hybrid films turned dark in color, suggesting that Ti^3+^ was successfully generated. The UV–Vis spectrum of these hybrid films after UV irradiation is shown in Figure 4b, each film exhibited maximum absorption at approximately 600 nm derived from Ti^3+^. However, this absorption disappeared in each sample within approximately 10 days (Figure 4c and Figure S6). To evaluate the UV weatherability of each hybrid polymer, transmittance at 600 nm was monitored over time. As shown in Figure 4c, visible-light transmittance was restored in all the hybrid films. However, the restoration at 400 nm was incomplete (Figure S7). Moreover, the recovery rate did not depend on the composition of the matrix. Therefore, the Ti^3+^ generated using UV did not completely return to its original state. To investigate the effect of the polymer matrix on UV irradiation, FTIR measurements of these hybrid films before and after UV irradiation were carried out (Figure S8). The absorption bands at 1402–1450 cm^−1^, 2857–2925 cm^−1^, 1240 cm^−1^, 1530 cm^−1^, and 1740 cm^−1^ were assigned to the C–H bending, C–H stretching, C–O stretching, N–H bending, and C=O stretching of polycarbonates, respectively. The peak attributed to the main chain cannot be observed before and after UV irradiation [36,37,38,39]. This phenomenon is attributed to the absorption of the partial oxygen deficiency state of titania and not the coloration due to the modification of the polycarbonate matrix. 

Figure 4d shows the tensile test results of the hybrid films before and after UV irradiation. The Young’s modulus and fracture energy calculated from the tensile tests are summarized in Table S1. The tensile data of PC0 are not available because it was extremely soft and brittle. The Young’s modulus decreased as the ratio of the ring structure decreased, and this effect was correlated to the difference in *T*_g_ as measured using DMA. Furthermore, material elongation did not change linearly with the ratio of the ring structure. Moreover, the fracture energy was maximized when the ring structure ratio was 50% due to the increased elongation of the material. These results suggest that the ring structure and the chain structure were phase-separated, although they were copolymerized in the matrix. In other words, the ring structure probably aggregated partially in the matrix and functioned as a hard segment. After UV irradiation, none of the samples exhibited significant changes in the tensile data (Figure 4d, dotted line). Hence, the generation of hydroxyl radicals accompanying the formation of Ti^3+^ did not affect the polymer matrix, which supports the hypothesis that the absorption of 400 nm is not associated with the denaturation of the polymer matrix.

### 3.3. Lifetime of Ti^3+^, Oxygen Permeability, and Magnetic Properties

**Figure 5:**
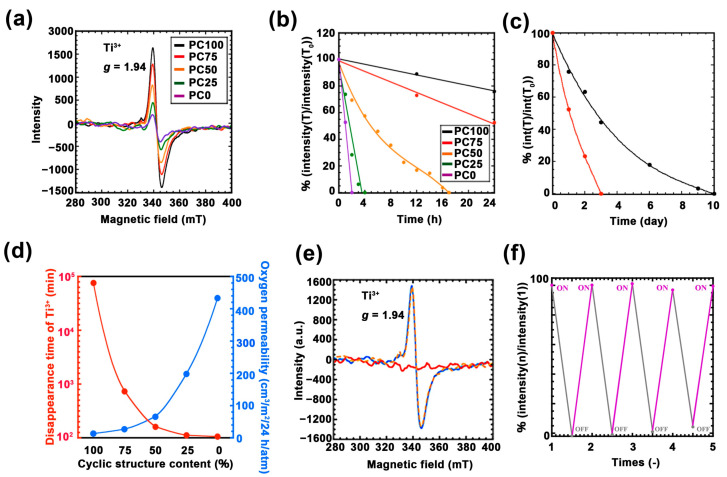
(**a**) Electron spin resonance (ESR) spectra for each UV irradiated the polycarbonate/titania hybrid films after 1 h: PC100 (black line), PC75 (red line), PC50 (orange line), PC25 (green line), and PC0 (purple line). (**b**) Time variation of normalized ESR intensity of each UV irradiated hybrid film with hour scale: PC100 (black line), PC75 (red line), PC50 (orange line), PC25 (green line), and PC0 (purple line). (**c**) Time variation of normalized ESR intensity of each UV irradiated hybrid film with day scale: PC100 (black line) and PC75 (red line). (**d**) Correlation diagram of each hybrid film of lifetime of Ti^3+^ (red line) and oxygen permeability (blue line). (**e**) ESR spectra for PC100 after each treatment: UV irradiation after 2 h (blue line), UV irradiation after 12 h (orange dotted line), and UV irradiation after 2 h and heating treatment (red line). (**f**) Repeated UV irradiation test of PC100: after 80 °C incubation for 2 h (black plot), and after UV irradiation for 2 h (purple plot).

To quantitatively evaluate the amount of photo-induced Ti^3+^ in each hybrid film, ESR measurements were performed. Figure 5a shows the ESR spectra of each film immediately after UV irradiation. In all the films, the topmost region of the peak can be confirmed at *g* = 1.94, which is derived from Ti^3+^ [40,41,42]. The intensity of this peak increased as the ring structures increased. Considering the oxygen permeability data, this result suggested that the formation and disappearance of Ti^3+^ are in an equilibrium. Therefore, the maximum amount of Ti^3+^ obtained through UV irradiation was limited by the oxygen permeability of the hybrid film. Next, to measure the lifetime of Ti^3+^ generated during UV irradiation, the ESR intensity of Ti^3+^ peak against time was profiled (Figure S9). Figure 5b,c shows the normalized ESR intensity ratio versus time. The lifetime of Ti^3+^ was prolonged as the ratio of the ring structure increased. Between the samples with the shortest Ti^3+^ lifetime (PC0, 120 min) and the longest lifetime (PC100, 14,400 min), the difference was approximately 120 times. Further, Figure 5d summarizes the disappearance time of Ti^3+^, and oxygen permeation, exhibiting a strong correlation among the two. Thus, the lifetime of Ti^3+^ depends on the oxygen permeability. 

Furthermore, to investigate changes in the lifetime of Ti^3+^ and glass transition temperature PC100 (which exhibited the longest Ti^3+^ lifetime) was incubated at a temperature higher than its *T*_g_ (80 °C), and the disappearance of Ti^3+^ was measured using ESR (Figure 5e). The ESR intensity derived from Ti^3+^ completely disappeared after 2 h of incubation above *T*_g_, whereas it exhibited no change when the sample was incubated at room temperature for 12 h. Furthermore, our previous study has reported Ti^3+^ that is generated in the poly(methyl methacrylate) (PMMA)-titania hybrid system, which featured a higher Tg than PC100. It completely disappeared in one day at room temperature [43]. It can be concluded that heating the hybrid film above *T*_g_ eliminated the packing structure that is important for oxygen tightness. Furthermore, we confirmed the repeated generation of Ti^3+^ for PC100 using ESR (Figure 5f). In this experiment, Ti^3+^ was generated through UV irradiation, the material was heated to 100 °C to eliminate Ti^3+^, and the generation of Ti^3+^ through UV irradiation was repeated five times. As a result, it became evident that Ti^3+^ was repeatedly generated. These results indicate the applicability of this material, which can quickly and repeatedly change Ti^3+^ and Ti^4+^ in combination with heat treatment. 

**Figure 6:**
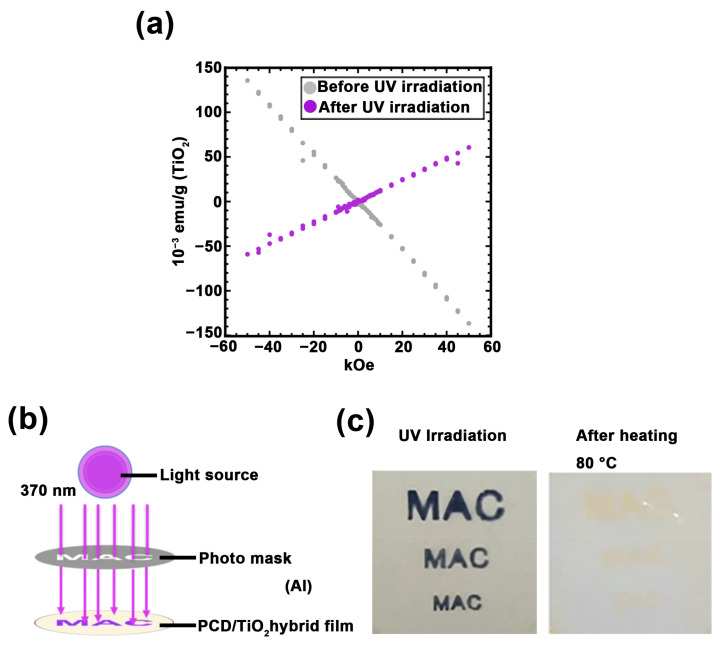
(**a**) Superconducting quantum interference device (SQUID) magnetometer measurements of PC100: before UV irradiation (gray plot) and after UV irradiation (purple plot). (**b**) The image of photolithography of PC100 by UV light source. (**c**) Photograph of PC100 with photolithography and heating treatment.

Further, the magnetic properties of Ti^3+^ generated in PC100 were examined using SQUID (Figure 6a). It was confirmed that the sample that was not irradiated with UV exhibited diamagnetic properties, whereas the sample irradiated with UV exhibited paramagnetic properties. Moreover, partially changing the magnetic susceptibility rather than as a whole magnetic susceptibility of material has the potential to expand applications, such as magnetic storage media. In addition, the ability of the PC100 with canceling paramagnetism by heating is considered to be one of the useful property for managing storage media. It was demonstrated that Ti^3+^ were generated in selected regions using a photomask, and it disappeared after heating (Figure 6b,c). Moreover, ESR results demonstrate that these operations can be used repeatedly without compromising Ti^3+^ production efficiency (Figure 5f). Therefore, these results exhibit the attractive magnetic properties of PC100.

## 4. Conclusions

Titania hybrid films were successfully prepared using the sol–gel method, starting from an alkoxysilane end-modified polycarbonate oligomer with different ratios in the ring structure. All the hybrid polymers exhibited high visible-light transmission, which was maintained after Ti^3+^ generation under UV irradiation. The ring structure in the polymer matrix contributed to the oxygen-barrier properties due to its enhanced packing force. Tensile tests confirmed that the hybrid polymers resisted changes in the mechanical properties of their polymer matrices under UV irradiation, and the copolymerized ring structure functioned as a hard segment due to phase separation. The lifetime of the generated Ti^3+^ depended on the oxygen-barrier properties of the polymer matrices and were extended by a factor of up to 120. Photolithography using UV light facilitated the local switching of the hybrid film between paramagnetism and diamagnetism. The polymer matrices reported herein possess both UV-weather resistance and controllable oxygen permeability, and they will be useful in the design of oxygen-sensitive inorganic materials or inorganic complexes that are produced by photoreaction.

## Figures and Tables

**Table 1:** Summary of the precursor and sample names.

Precursor Name	3-PTES Modified	Sample Name (40 wt% Titania)	Percentage of Ring Structure (%)
PHMCD	ET-PHMCD	PC0	0
coPCD 13	ET-coPCD 13	PC25	25
coPCD 11	ET-coPCD 11	PC50	50
coPCD 31	ET-coPCD 31	PC75	75
PCDCH	ET-PCDCH	PC100	100

**Table 2:** Distance between titania domains in each hybrid polymer obtained by SAXS measurement.

Samples Name	PC0	PC25	PC50	PC75	PC100
Distance of titania domain (nm)	5.71	4.87	4.59	4.59	4.55

## Supplementary Materials

The following are available online at https://www.mdpi.com/2079-4991/11/1/5/s1, Figure S1: 1H-NMR spectrum of ET-PHMCD, Figure S2: ^1^H-NMR spectrum of ET-coPCD 31, Figure S3: ^1^H-NMR spectrum of ET-coPCD 11, Figure S4: ^1^H-NMR spectrum of ET-coPCD 13, Figure S5: ^1^H-NMR spectrum of ET-PCHCD, Figure S6: UV-Vis spectra of different hybrid films 10 days after UV irradiation: PC100 (black),PC75 (red), PC50 (orange), PC25 (green), and PC0 (purple).Figure S7: Transmittance of different hybrid films at 400 nm: before UV irradiation, immediately after 2 h of UV irradiation, and 10 days after irradiation. PC100 (black), PC75 (red), PC50 (orange), PC25 (green), and PC0 (purple). Figure S8: FT-IR spectral profiles of hybrid films: before UV irradiation (red line), UV irradiation after 12 h (orange line), (a) PC100, (b) PC75, (c) PC50, (d) PC25, and (e) PC0.Figure S9: ESR spectral profiles of hybrid films after UV irradiation: (a) PC100, (b) PC75, (c) PC50, (d) PC25, and (e) PC0. Table S1: Summary of results from the tensile test and DMA measurement.
