# Supplementary material for: Polycarbonate/Titania Hybrid Films with Localized Photo-Induced Magnetic-Phase Transition
Source: Nanomaterials (Basel). 2020 Dec 22;11(1):5. doi: 10.3390/nano11010005 (PMC7822203; doi:10.3390/nano11010005)
Supplement: Supplementary file 1 [file nanomaterials-11-00005-s001.pdf]

nanomaterials

Supplementary Materials

Polycarbonate/Titania Hybrid Films with Localized Photo-Induced Magnetic-Phase Transition

Shuta Hara, Sei Kurebayashi, Genza Sanae, Shota Watanabe, Takehiro Kaneko, Takeshi Toyama, Shigeru Shimizu and Hiroki Ikake *

Department of Materials and Applied Chemistry, College of Science and Technology, Nihon University, 1-8-14 Kandasurugadai, Chiyoda-ku, Tokyo 101-8308, Japan; hara.shuta@nihon-u.ac.jp (S.H.); sei.kurebayashi@polymer.chem.cst.nihon-u.ac.jp (S.K.); genza.sanae@polymer.chem.cst.nihon-u.ac.jp (G.S.); syouta.watanabe@polymer.chem.cst.nihon-u.ac.jp (S.W.); kaneko.takehiro@nihon-u.ac.jp (T.K.); touyama.takeshi@nihon-u.ac.jp (T.T.); shimizu.shigeru@nihon-u.ac.jp (S.S.)

* Correspondence: ikake.hiroki@nihon-u.ac.jp; Tel./Fax.: +81-(3)- 3259-0823

Supporting Figures

Figure S1. ^{1}H-NMR spectrum of ET-PHMCD.
Figure S2. ^{1}H-NMR spectrum of ET-coPCD 31.
Figure S3. ^{1}H-NMR spectrum of ET-coPCD 11.
Figure S4. ^{1}H-NMR spectrum of ET-coPCD 13.
Figure S5. ^{1}H-NMR spectrum of ET-PCHCD.

Figure S6. UV-Vis spectra of different hybrid films 10 days after UV irradiation: PC100 (black),PC75 (red), PC50 (orange), PC25 (green), and PC0 (purple).

Figure S7. Transmittance of different hybrid films at 400 nm: before UV irradiation, immediately after 2 h of UV irradiation, and 10 days after irradiation. PC100 (black), PC75 (red), PC50 (orange), PC25 (green), and PC0 (purple).

Figure S8. FT-IR spectral profiles of hybrid films: before UV irradiation (red line), UV irradiation after 12 h (orange line), (**a**) PC100, (**b**) PC75, (**c**) PC50, (**d**) PC25, and (**e**) PC0.

Figure S9. ESR spectral profiles of hybrid films after UV irradiation: (**a**) PC100, (**b**) PC75, (**c**) PC50, (**d**) PC25, and (**e**) PC0.

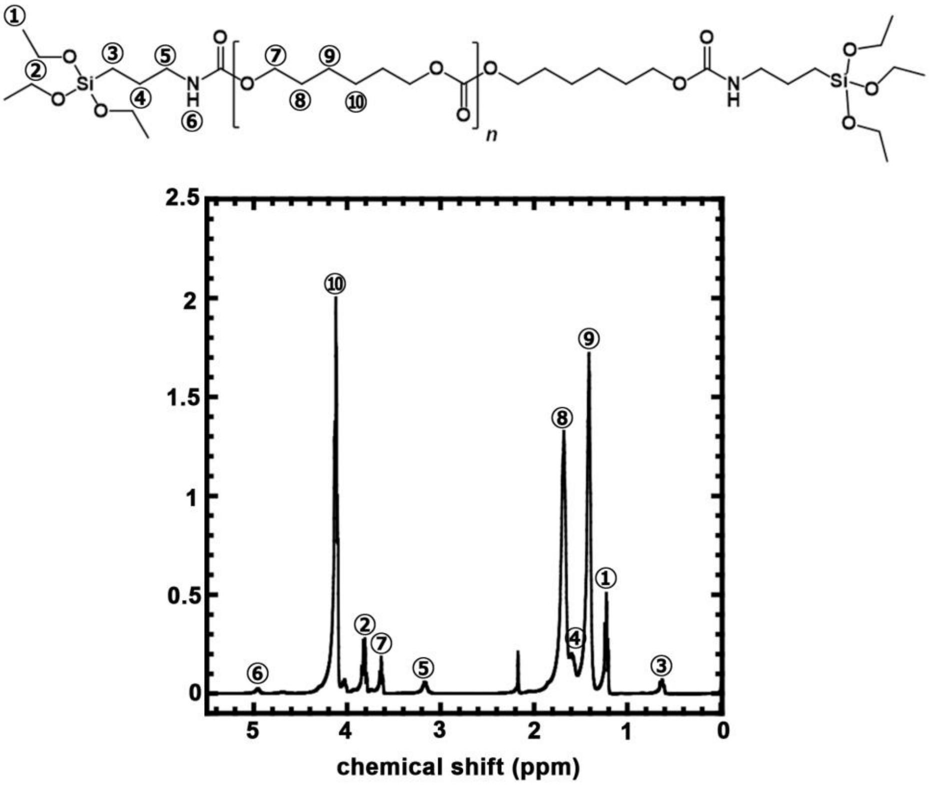

Figure S1. ^{1}H-NMR spectrum of ET-PHMCD.

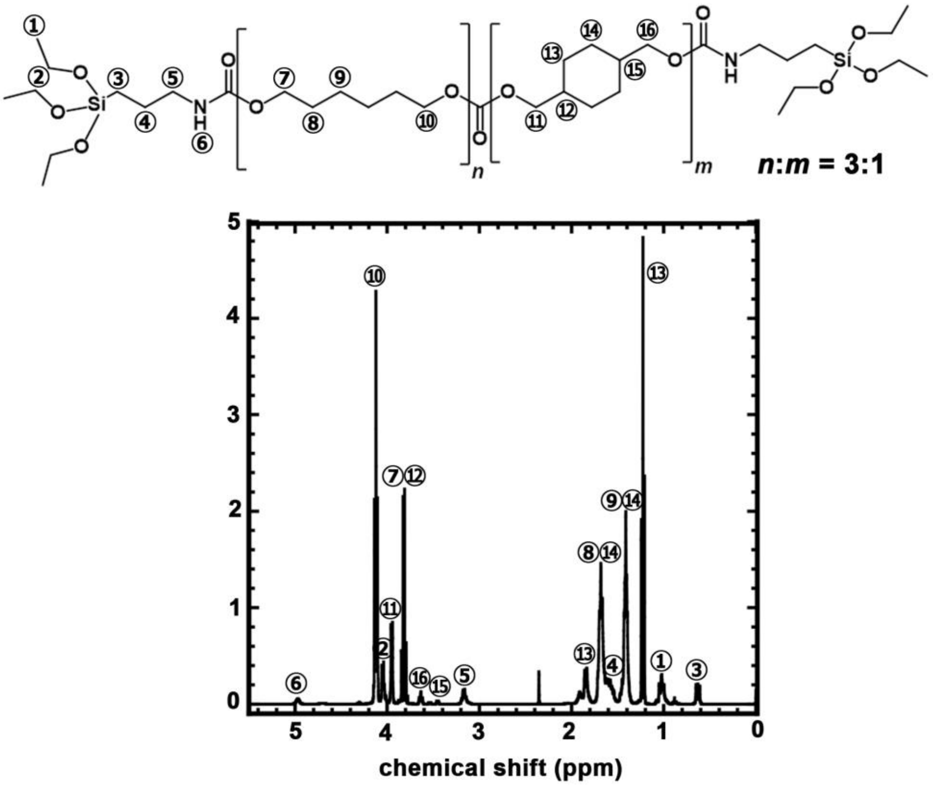

Figure S2. ^{1}H-NMR spectrum of ET-coPCD 31.

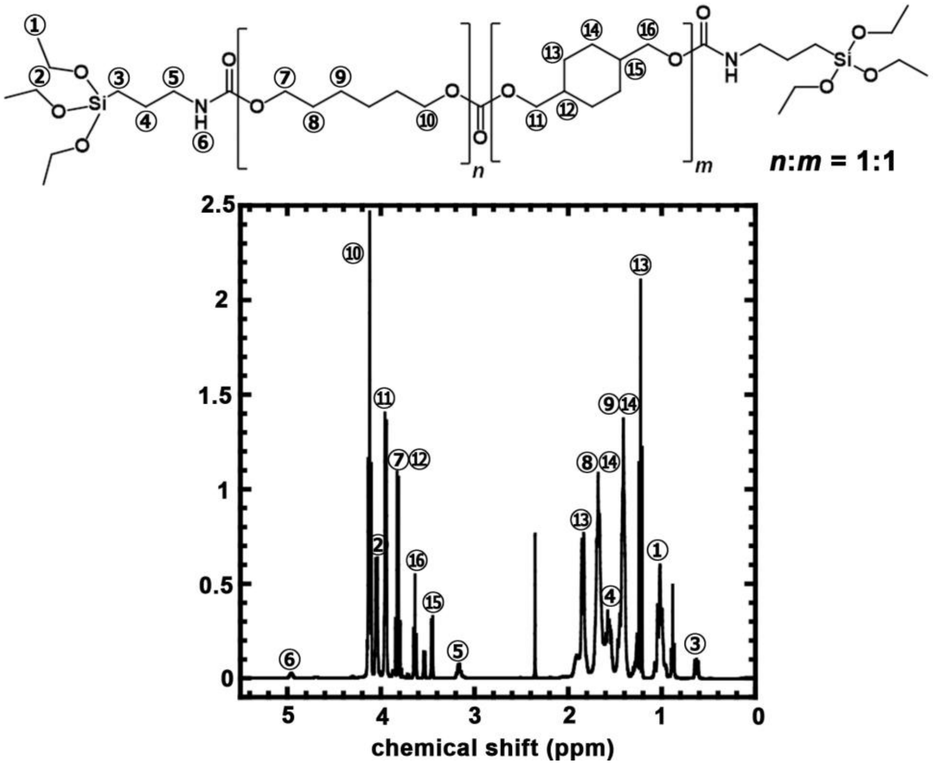

Figure S3. ^{1}H-NMR spectrum of ET-coPCD 11.

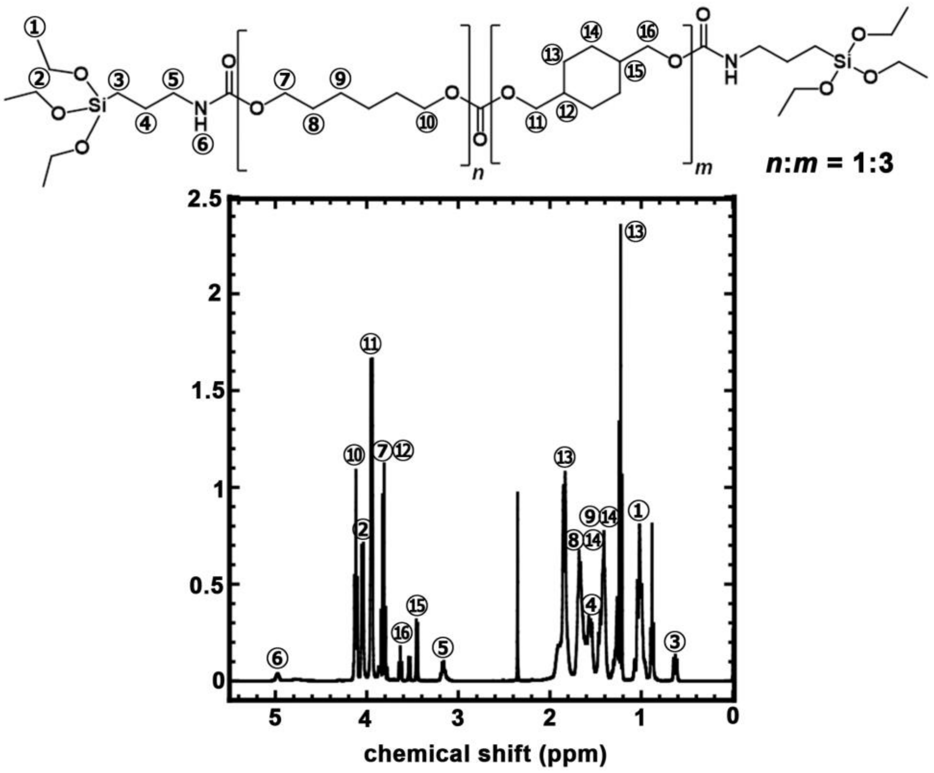

Figure S4. ^{1}H-NMR spectrum of ET-coPCD 13.

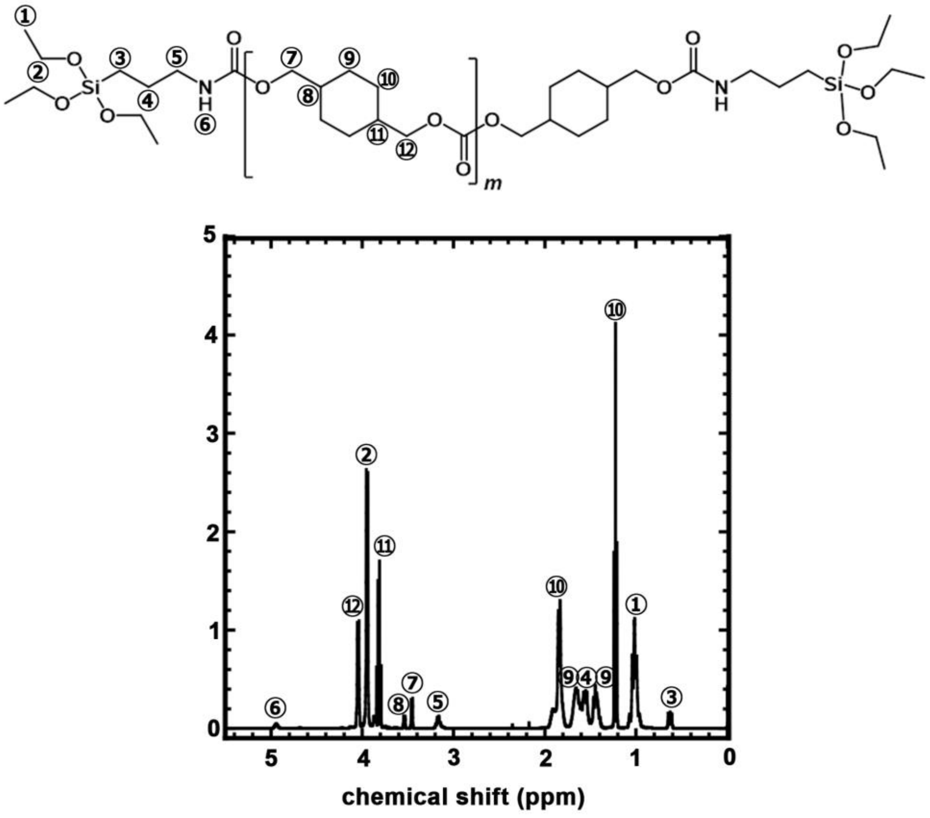

Figure S5. ^{1}H-NMR spectrum of ET-PCHCD.

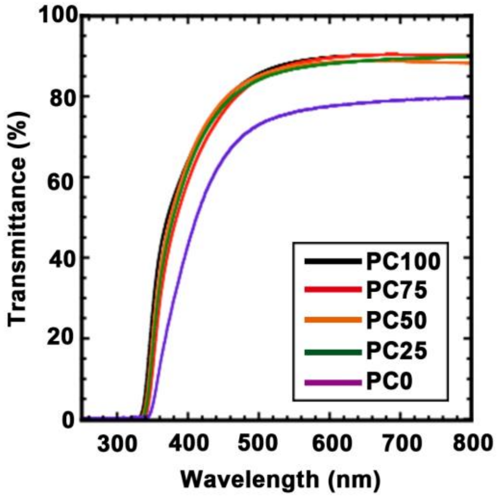

Figure S6. UV-Vis spectra of different hybrid films 10 days after UV irradiation: PC100 (black), PC75 (red), PC50 (orange), PC25 (green), and PC0 (purple).

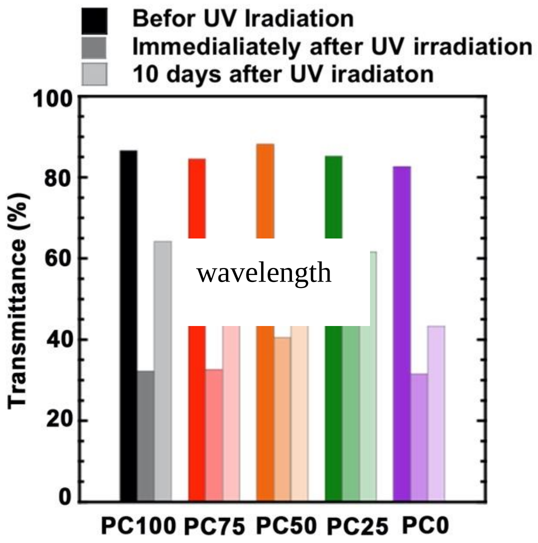

Figure S7. Transmittance of different hybrid films at 400 nm: before UV irradiation, immediately after 2 h of UV irradiation, and 10 days after irradiation. PC100 (black), PC75 (red), PC50 (orange), PC25 (green), and PC0 (purple).

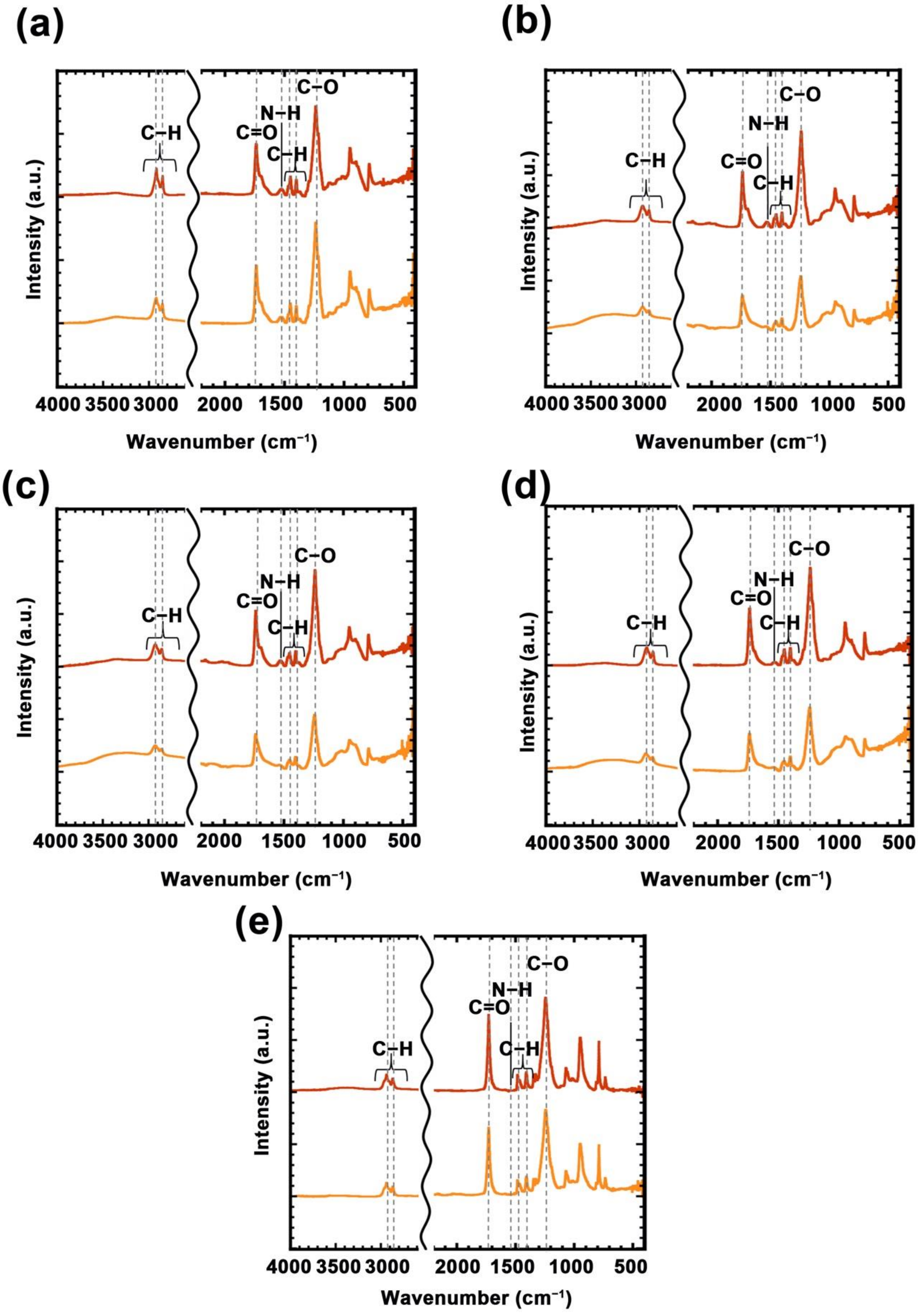

Figure S8. FT-IR spectral profiles of hybrid films: before UV irradiation (red line), UV irradiation after 12 h (orange line), (**a**) PC100, (**b**) PC75, (**c**) PC50, (**d**) PC25, and (**e**) PC0.

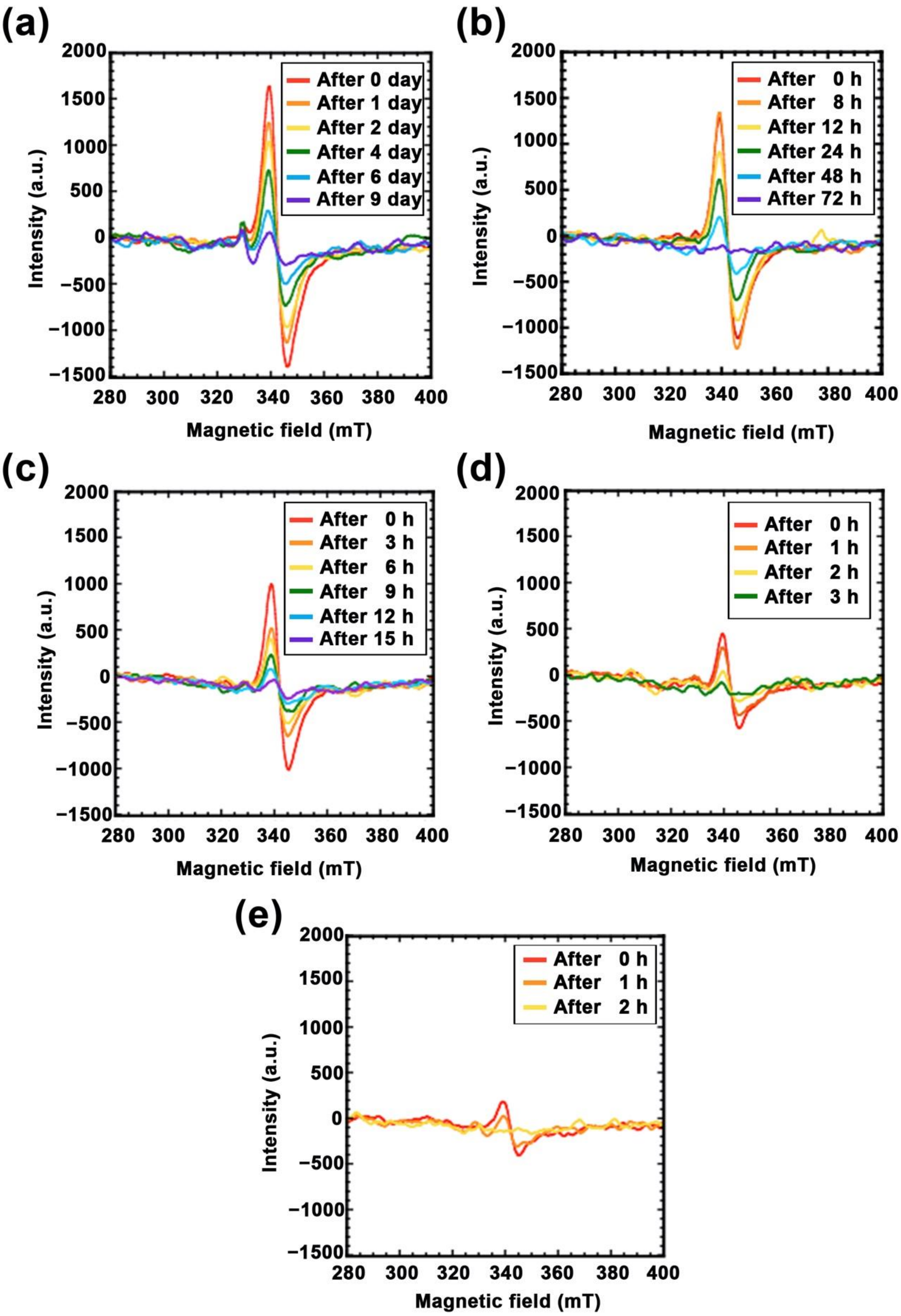

Figure S9. ESR spectral profiles of hybrid films after UV irradiation: (**a**) PC100, (**b**) PC75, (**c**) PC50, (**d**) PC25, and (**e**) PC0.

Table S1. Summary of results from the tensile test and DMA measurement.

Sample	Tensil strength	Young modulus	fracture energy	Storage E′at 25 °C	Tg (DMA)
	(MPa)	（GPa）	(MJ/m3)	E' (Pa)	(°C)
PC100	23.69	3.81	190.4	$2.36*10^9$	68.5
PC75	9.07	0.97	139.6	$1.54*10^9$	40.6
PC50	10.92	0.26	227.8	$3.45*10^7$	12.8
PC25	3.45	0.10	54.4	$1.94*10^7$	1.9
PC0	-	-	-	$1.91*10^7$	-28.8
